# Silica Inverse Opal Nanostructured Sensors for Enhanced Immunodetection of Extracellular Vesicles by Quartz Crystal Microbalance with Dissipation Monitoring

**DOI:** 10.1021/acsanm.2c02775

**Published:** 2022-08-19

**Authors:** Jugal Suthar, Alberto Alvarez-Fernandez, Alaric Taylor, Maximiliano J. Fornerod, Gareth R. Williams, Stefan Guldin

**Affiliations:** † Department of Chemical Engineering, University College London, Torrington Place, London WC1E 7JE, U.K.; ‡ UCL School of Pharmacy, University College London, Bloomsbury, 29-39 Brunswick Square, London WC1N 1AX, U.K.

**Keywords:** inverse opal, extracellular vesicles, sensing, QCM, colloids, co-assembly

## Abstract

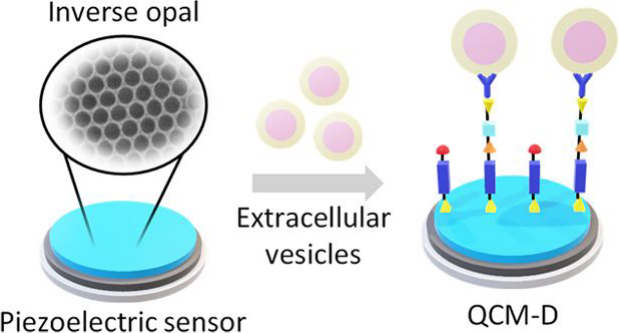

Extracellular vesicles (EVs) are nanosized circulating assemblies that contain biomarkers considered promising for early diagnosis within neurology, cardiology, and oncology. Recently, acoustic wave biosensors, in particular based on quartz crystal microbalance with dissipation monitoring (QCM-D), have emerged as a sensitive, label-free, and selective EV characterization platform. A rational approach to further improving sensing detection limits relies on the nanostructuration of the sensor surfaces. To this end, inorganic inverse opals (IOs) derived from colloidal self-assembly present a highly tunable and scalable nanoarchitecture of suitable feature sizes and surface chemistry. This work systematically investigates their use in two-dimensional (2D) and three-dimensional (3D) for enhanced QCM-D EV detection. Precise tuning of the architecture parameters delivered improvements in detection performance to sensitivities as low as 6.24 × 10^7^ particles/mL. Our findings emphasize that attempts to enhance acoustic immunosensing via increasing the surface area by 3D nanostructuration need to be carefully analyzed in order to exclude solvent and artifact entrapment effects. Moreover, the use of 2D nanostructured electrodes to compartmentalize analyte anchoring presents a particularly promising design principle.

## Introduction

Extracellular vesicles (EVs) are nanosized assemblies contained by a lipid bilayer membrane that are released from cells as part of their routine processing.^1−4^ Depending on the cell of origin, EVs are found to carry biomolecular content that is essential for intercellular communication and disease propagation, in the form of proteins, DNA, RNA, and lipids.^5,6^ Evidence now demonstrates that the detection and quantification of EVs can help to unravel pathological pathways for many diseases, particularly within neurology, cardiology, and oncology, emphasizing the necessity to establish sensitive, specific, and reproducible EV detection techniques.^7−9^ X-ray scattering,^10^ fluorescence spectroscopy,^11^ Raman spectroscopy,^12^ or electrochemical measurements^13^ are just some of the novel EV characterization approaches recently reported. However, in general, none of these techniques currently meets the combination of prerequisites on the detection limit, specificity, label-free recognition, and minimal sample volume. As a result, available approaches lack the sensitivity of detecting at clinically relevant biomarker concentrations; are unable to discern reliably between EV and non-EV artifacts with increasing risk of false-positive results; require diagnostic reagents for labeling that increase the assay complexity and cost; and/or are unsuitable for minimally invasive liquid biopsies because of the requisite sample volume.

A particularly promising analytical principle for multimodal biosensing is based on acoustic resonance.^14^ The application of a quartz crystal microbalance with dissipation monitoring (QCM-D) has been shown to uniquely leverage differences between EVs and associated contaminants in colloidal suspension by assessing both mass and viscoelastic properties, thus offering a superior level of analytical discrimination. The dual mechanisms of measurement offered by QCM-D helped to overcome the current limitations of specificity within the EV sensing field, providing an important addition to the characterization tool kit. Specifically, the immunocapture of CD63-positive EVs on gold-coated QCM sensor surfaces induces a change in resonance frequency of the QCM sensor because of the mass of the analyte. This was witnessed alongside a concomitant increase in dissipation, attributed to the soft, viscoelastic, nature of the EVs, which are bound as discrete particles that also undergo rocking and translational movement, incurring energy loss at the sensor surface.^15^ Such findings have built upon previous citations demonstrating surface acoustic wave detection of EVs that only offer a single mode of measurement.^16^

Despite the fact that QCM-D offers rich data on the build-up of functional interfaces, its limit-of-detection (LOD) remains relatively high (1.4 × 10^8^ particles/mL). Therefore, while the technique presents a valuable complementary tool for biosensor development, further improvements are required in terms of detection sensitivity to effectively integrate QCM-D within minimally invasive disease diagnostics. This was partly achieved by coupling the QCM-D assessment with tandem electrochemical impedance measurements via an EQCM-D-based detection platform (LOD 6.7 × 10^7^ particles/mL),^17^ but further efforts are needed.

An alternative route to further improve analytical sensitivity and specificity in QCM-D is via nanostructuration of the electrode surface, with the rationale being to increase the sensing surface area (surface-to-volume ratio) for enhanced binding capacity and/or to modify the aspect ratio (length to diameter ratio) for optimal ligand arrangement. Da Kyeong Oh and co-workers showed improved specificity, faster kinetics, and higher sensitivity with the introduction of 2D and 3D molecularly imprinted polymers on 2D inverse opals (IOs) for the recognition of Bisphenol A and macromolecular proteins, respectively.^18,19^ Other nanoarchitectures such as anodic aluminum oxide or ZnO nanotips have been used to increase the surface area of the QCM-D sensor against different targets (such as enzymes,^20^ liposomes,^21^ and antibodies^22^) or for enhanced cell adhesion and proliferation.^23^

In this context, inorganic IOs derived by colloidal self-assembly present a highly tunable and scalable nanoarchitecture of suitable feature sizes and surface chemistry.^24−26^ IOs are three-dimensional porous structures with a regular arrangement of interconnected spheroid cavities that have a large internal surface area and a uniformity in the pore size (ranging between 100 and 1000 nm) on the macroscale.^27^ These properties make them ideal candidates for a myriad of applications, such as in catalytic systems,^28,29^ photonics,^30,31^ electrochemistry, and energy devices.^32−34^ 3D inverse opals (3D IOs) may be fabricated using top-down techniques such as photo- and electron beam lithography or nanoimprinting,^35,36^ or via bottom-up techniques such as colloidal assembly with sacrificial spheres.^30,37^ One particularly attractive route is by co-assembly, where an inorganic sol–gel precursor is added to a colloidal suspension and therefore participates in an evaporative self-assembly process at the meniscus of a substrate. The result is minimized cracking and inhomogeneities associated with the multistep process of standard colloidal assembly.^27,38^ While colloids offer precise control over porous networks on the 100 nm to micrometer length scale, co-assembly techniques involving block copolymer micelles are particularly suited for pore diameters below 100 nm.^39−43^

The establishment of such precise and facile manufacturing methods for 3D IOs has facilitated their integration into biosensing.^44^ The enhanced surface area offered by the incorporation of 3D IO structures, in combination with their optical properties, has been successfully exploited for improving the analytical performance of multiple sensing platforms. To this end, Li and co-workers developed a label-free biosensor based on TiO_2_ inverse opal films and reflectometry interference spectroscopy.^45^ The physical adsorption of proteins on the pore surface was monitored by the shift in the reflection peak, allowing detection limits as low as 1 μg mL^–1^. Following a similar approach, Lee et al. successfully immobilized antibodies onto silica 3D IO nanostructures to create a label-free optical immunosensor capable of detecting influenza viruses with high sensitivity (10^3^–10^5^ plaque-forming units) and specificity.^46^ Other examples of IO-based biosensors include an immunosorbent assay built on an amylase-based enzymatic 3D IOs^47^ and a DNA sensor based on the immobilization of fluorescent aptamers to 3D IO silica structures.^48^ Of closer relevance to the work described herein, Dong et al. created gold-coated TiO_2_ 3D IOs to successfully capture EVs and obtain spectroscopic information from bonds within exosomal phosphoproteins, enabling specific differentiation between EVs isolated from cancer patients and healthy individuals.^49^ However, the application of nanostructured surfaces for QCM-D-based EV biosensing remains unexplored. Moreover, solvent and artifact entrapment effects on the 2D and 3D nanostructured sensing surfaces have been constantly disregarded, preventing their full validation for real-world applications.

In response, this work explores the formation of IO porous structures atop QCM-D silica sensors for EV detection. Through the optimization of two different colloidal co-assembly methods (a vertical withdrawal and an evaporative deposition technique), FCC-structured silica IOs without cracks and low defect density were successfully formed in 2D and 3D. Scanning electron microscopy (SEM) and grazing incidence small-angle X-ray scattering (GISAXS) were used to confirm the structural properties of the created structures, including the layer thickness, pore size, and porosity. The impact of these parameters on detection sensitivity was subsequently investigated on a QCM-D platform, following silane-based functionalization of the silica surfaces and immunocapture of CD63-positive EVs in complex media. Evaluation of detection limits for mono- and multilayer IOs and flat silica surfaces was achieved by determining their contributions to background (nonspecific) signals.

## Results and Discussion

### Size Exclusion Chromatography Isolation of EVs from Cell Culture Media

**Figure 1:**
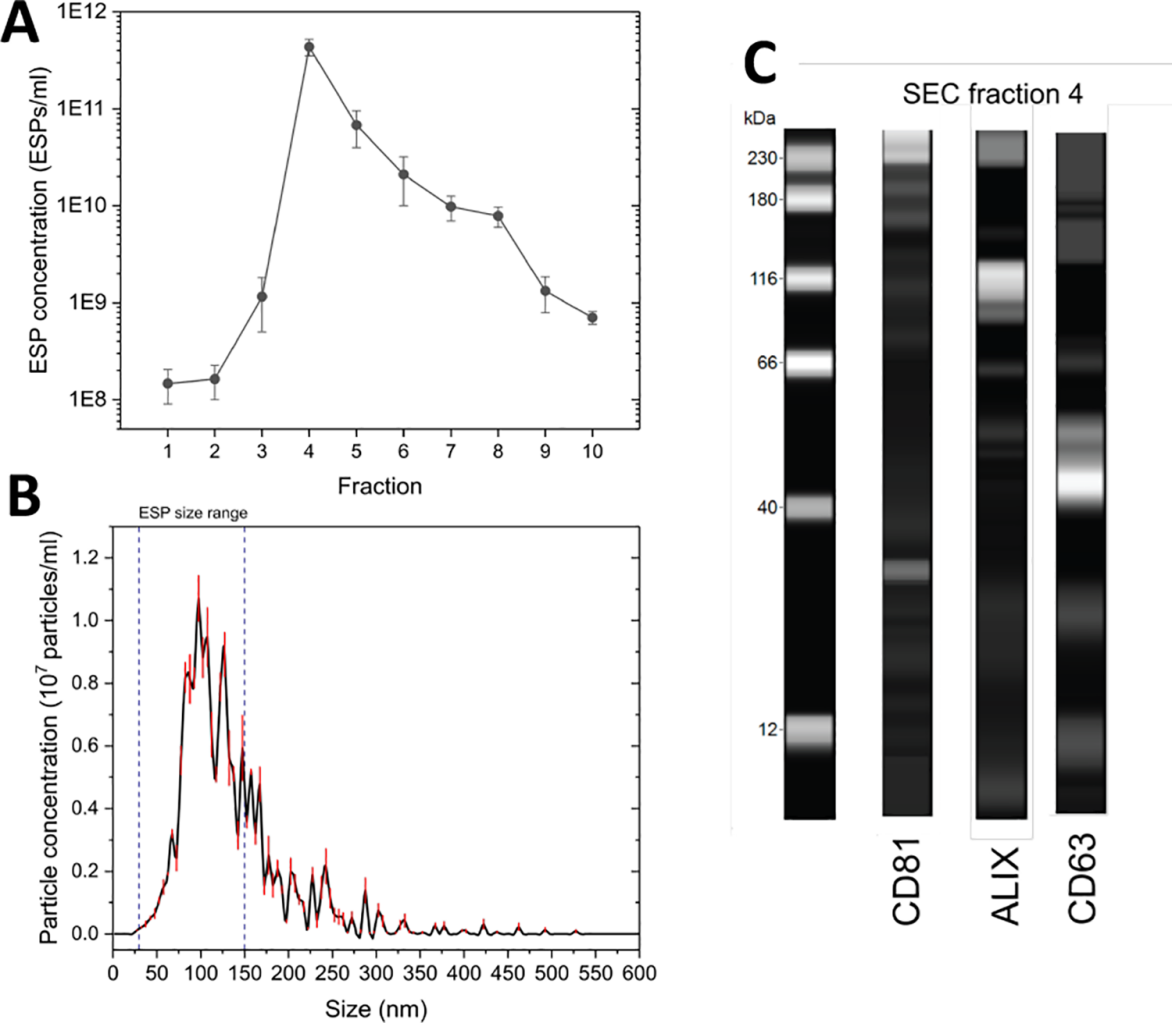
Concentration and particle size characterization of ESP obtained via a SEC protocol. (A) ESP concentration of SEC fractions. Standard deviation determined from three independent experiments. (B) Particle size distribution of SEC fraction 4. (C) Capillary gel-based electrophoresis western blot of SEC fraction 4, identifying EV enriched proteins, CD81 (26 kDa), Alix (93 kDa), and CD63 (57 kDa).

Prior to the detection of EVs on silica substrates, effective isolation from cell culture media was achieved through the implementation of a size exclusion chromatography (SEC) protocol. Nanoparticle tracking analysis (NTA) of the 10 eluted SEC fractions identified fraction 4 as possessing the highest concentration of EV-sized particles (ESPs) per mL. Concentrations of ESPs reduced steadily in subsequent fractions (Figure 1A). This designated fraction 4 for further analysis and confirmed EV presence. The size distribution profile of the particles in fraction 4 confirmed >91% of particles as being ESPs, with a modal size of 98 nm (Figure 1B). Western blot analysis successfully identified enriched exosomal proteins, namely, CD81, Alix, and CD63 (Figure 1C). This confirmed not only EV presence among the ESPs but also that the vesicles possessed good biological integrity. Moreover, it ensured that CD63 was in sufficient abundance to be used as the target protein molecule for subsequent immunodetection.

### Inverse Opal Structure Formation

**Figure 2:**
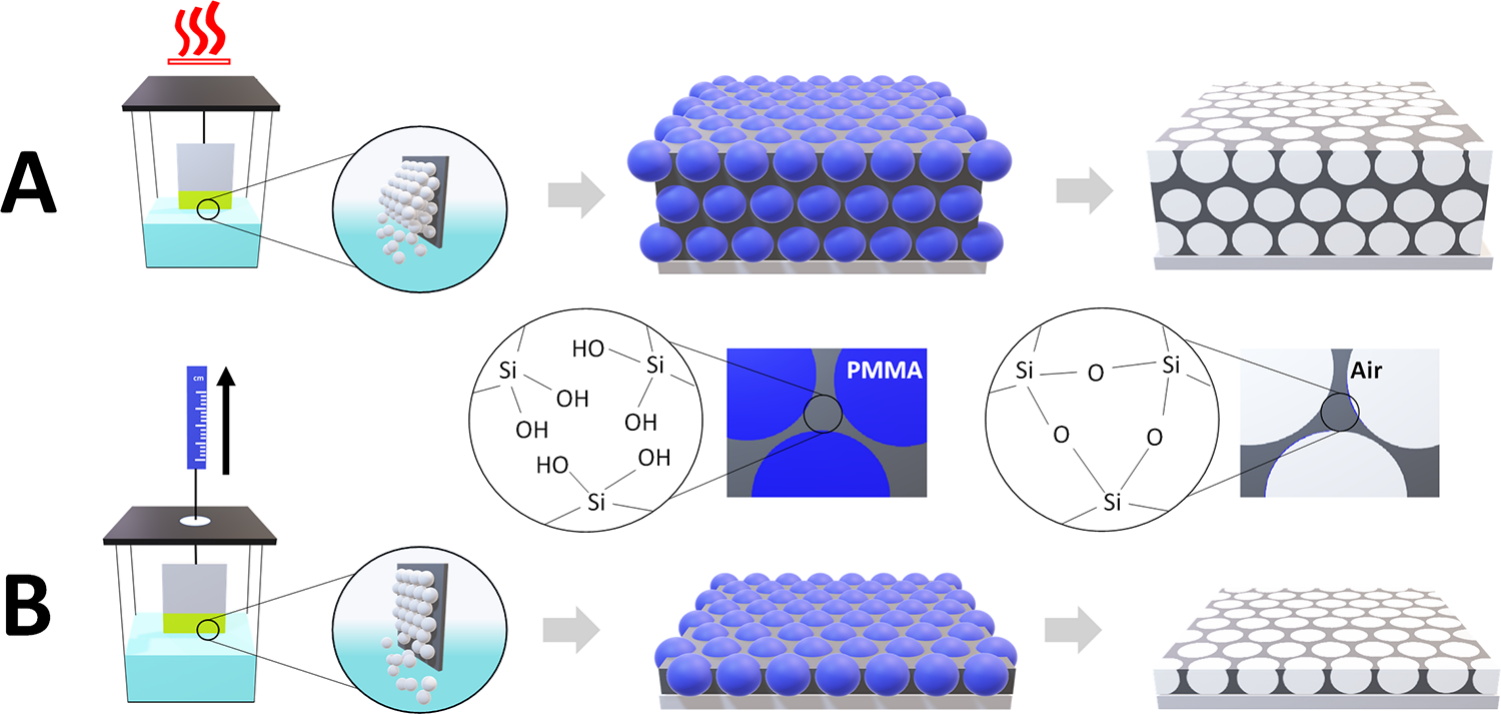
Schematic representation of implemented colloidal co-assembly methods and resulting inverse opal structures. (A) Evaporative deposition for multilayered IO formation approach. (B) Vertical withdrawal for monolayer formation.

IO structures with different pore sizes and thicknesses were obtained following the methodology illustrated in Figure 2. As a first step, co-assembly of poly(methyl methacrylate) (PMMA) colloidal spheres (of two different diameters: 250 and 600 nm, respectively) along with a silicate containing sol–gel solution was achieved using two approaches: vertical withdrawal (for 250 nm diameter spheres) and evaporative deposition (600 nm spheres). The sample holder apparatus for both techniques is displayed in Figure S1. Vertical withdrawal involved immersing a silica-coated substrate in the co-assembly mixture at room temperature. The slow withdrawal of the substrate at 0.01 mm/min created capillary forces at the meniscus that drive the assembly of the spheres and entrapment of the sol–gel matrix (hydrolyzed tetraethyl orthosilicate) in between. Evaporative deposition used elevated temperatures to initiate assembly. After the deposition was complete (the substrate withdrawn or mixture evaporated), opal structures (crystals) were subject to O_2_ ion etching (post-vertical withdrawal) or calcination (post-evaporative deposition) to remove the PMMA colloidal spheres and reveal the inorganic SiO_2_ IO network of pores. The rationale for selecting 600 nm-sized PMMA spheres for multilayer IOs was to provide sufficient pore and neck size for ESPs to infiltrate deeper within the porous network.

### Inverse Opal Structure Characterization

**Figure 3:**
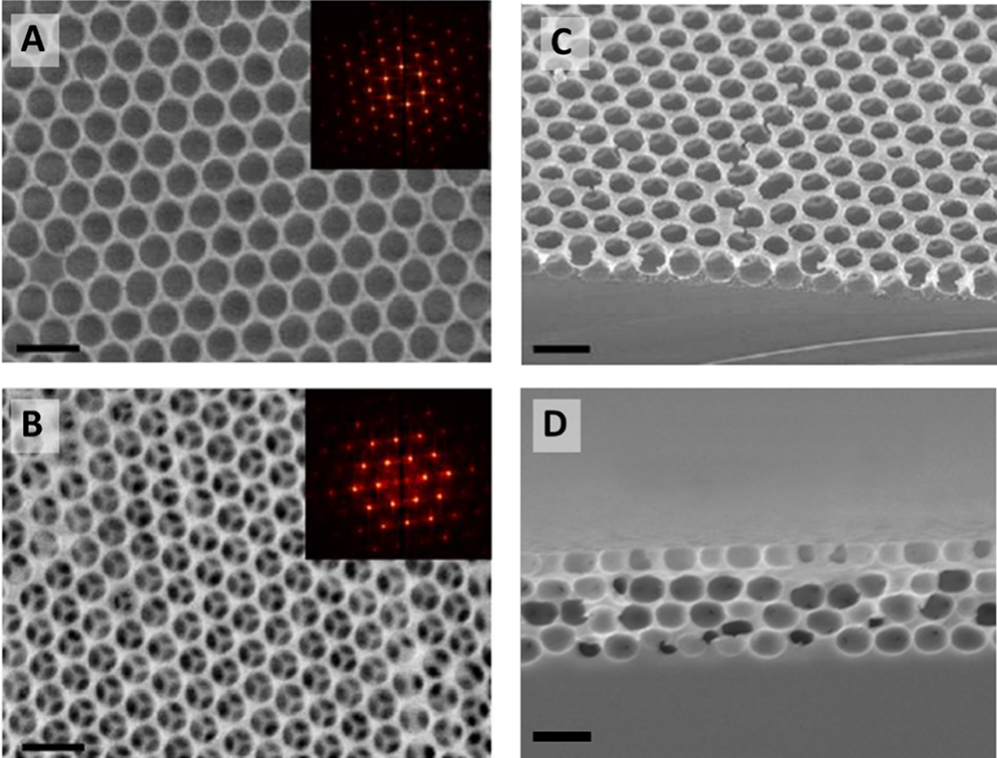
Top-view SEM micrographs of (A) inverse opal monolayer (scale bar: 400 nm) and (B) inverse opal multilayer (scale bars: 1 μm). Insets: FFT of respective SEM image. Cross-section SEM images of (C) monolayer and (D) multilayer IO architectures.

Top-view SEM images of the IO structures obtained after polymer removal show in both cases a crack-free and homogeneous porous structure. IOs produced via the vertical withdrawal method present a monolayer configuration, with excellent ordering as confirmed by FFT analysis (Figure 3A,C). This type of surface could provide the analyte with direct access to the underlying substrate and ensure that bound analytes are kept at a close distance to the oscillatory surface. IOs structures fabricated following the evaporative deposition approach produced a multilayer. The IO structure displayed a thickness of approximately 1500 nm (Figure 3B, D). The geometrical architecture of the close-packed pore structure could increase the tortuosity for analytes to reach the sensor surface and provide a greater internal surface area for immune-functionalization. The structural order of the 3D IO was further confirmed by small-angle X-ray scattering, displaying a face-centered cubic (FCC) structure with the {111} plane being parallel to the surface.^50−52^ Furthermore, films were seen to grow along the {110} direction of the deposited FCC structure (Figure S2).

Image analysis of the top-view SEM images (via the software Pebbles)^53^ enabled the calculation of average pore size distribution and total porosity in both structures. Monolayer IO pores were found to have a modal pore size of 247 ± 3 nm, which suggests minimal shrinkage from the original 250 nm PMMA sphere size during the ion etching process (Figure S3A). Multilayer IO pores displayed an estimated surface diameter of 494 ± 5 nm, which confirmed significant (17.7%) shrinkage of pores compared to the original 600 nm PMMA sphere size during the calcination process (Figure S3B). This is in line with previous studies and may be linked to the concurrent volume shrinkage of PMMA spheres and sol–gel precursors during the condensation reaction upon heating.^54,55^

Pore size analysis of the cross-sectional SEM image for multilayer IO suggests that the shrinkage of the pores occurred with a directional bias, with the modal height being 381 ± 4 nm, representing a 36.5% shrinkage (Figure S3C). The full pore width by comparison was determined to be 501 ± 3 nm, marking a 16.5% shrinkage and highlighting significant pore anisotropy to give an oblate ellipsoid (Figure S3D). In terms of total porosity, values of 73.1% (IO monolayer) and 64.7% (3D IO) were calculated. Both results are in line with conventionally reported porosity values for FCC-structured inverse opals.^27^

In a subsequent step, the internal surface area of both the IO monolayer and multilayer was estimated using previous structural information. The internal surface area for a single oblate ellipsoidal pore within the multilayer structure can be calculated following eq 1.
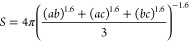
1where *S* is the surface area, *a* and *b* represent the in-plane radial dimensions, and *c* being the out-of-plane dimension from ellipsoid center to its surface.

Using structural values previously calculated by SEM, the total internal surface area for multilayer IO films was established at ∼215 μm^2^, marking a 43-fold increase in the surface area compared to a 5 μm^2^ flat silica surface. A similar calculation for the monolayer IOs was also made, although a correction was applied by halving the figure to account for the hemispherical shape of the obtained open porous monolayer. In comparison, the equivalent total internal surface area of the pores formed from the smaller 250 nm spheres was estimated to be ∼9.9 μm^2^, that is, a doubling of the surface area. This underlines the scope of IO structures for a significant increase in the detection surface area.

### 3DIO Modified Sensors for QCM-D Detection of EVs

**Figure 4:**
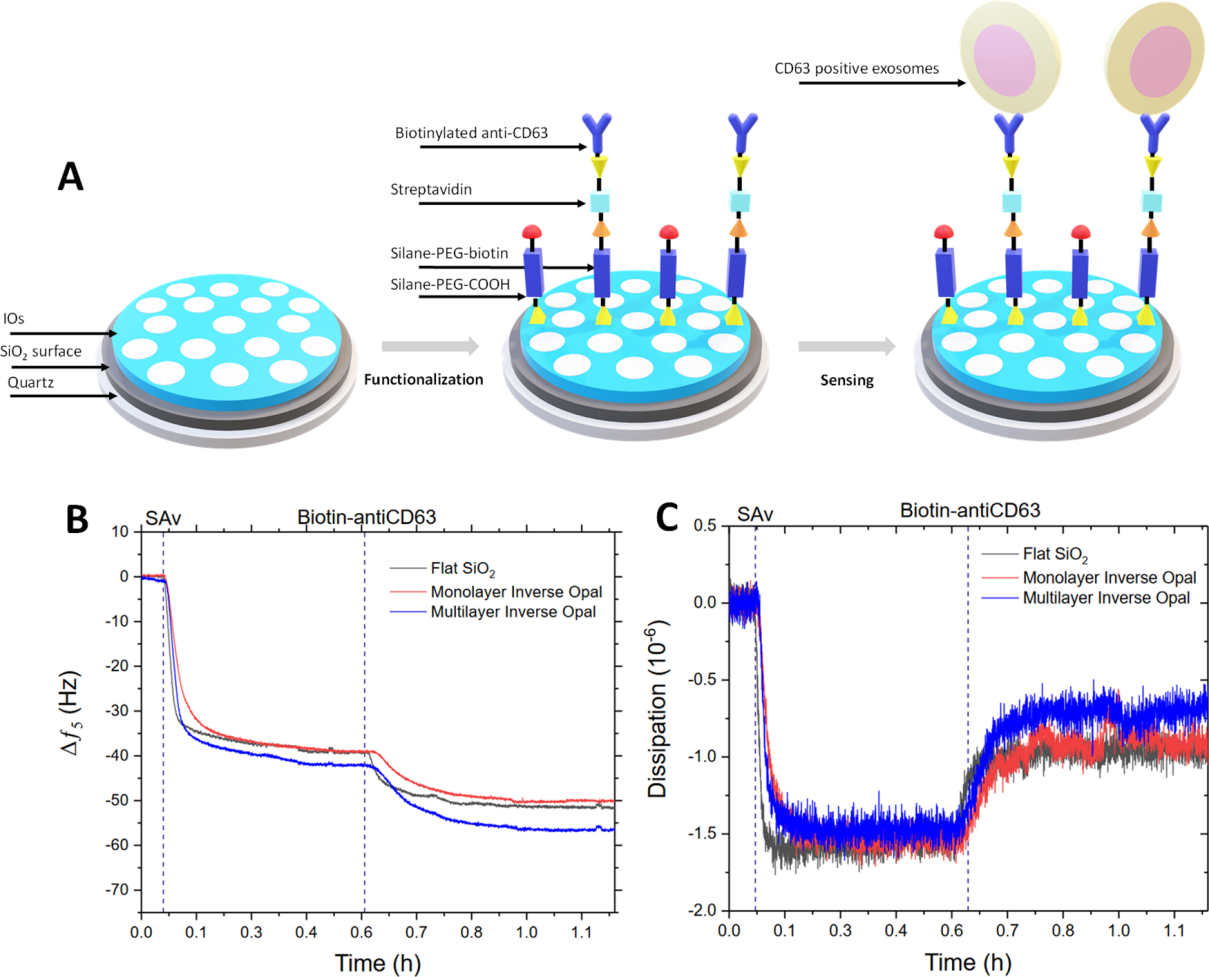
(A) Schematic representation of immunosensor fabrication atop IO coated sensors. QCM-D frequency (B) and dissipation (C) profiles captured for the immunosensor fabrication process on flat silica substrates compared with the IO monolayer and multilayer.

IO structures were applied to QCM sensors to understand the impact on the detection of ESP using the QCM-D platform and an immunosensing method, following the methodology displayed in Figure 4A. Silane-based chemistry was initially used to functionalized flat, monolayer IO and multilayer IO silica surfaces with a mixed-SAM that presents biotin molecules for subsequent streptavidin (Sav) conjugation. The QCM-D response to the fabrication process is shown in Figure 4B, C, confirming the adsorption of the relevant immuno-detection layers. The small magnitude of frequency and dissipation response is in line with the low mass and rigid nature of the proteins and with previously reported responses.^14^ It is important to note that while the additional decrease of frequency for multilayer inverse opals of ∼6 Hz (or ∼12%) is evident, the extend does not scale linearly with the surface area.

**Figure 5:**
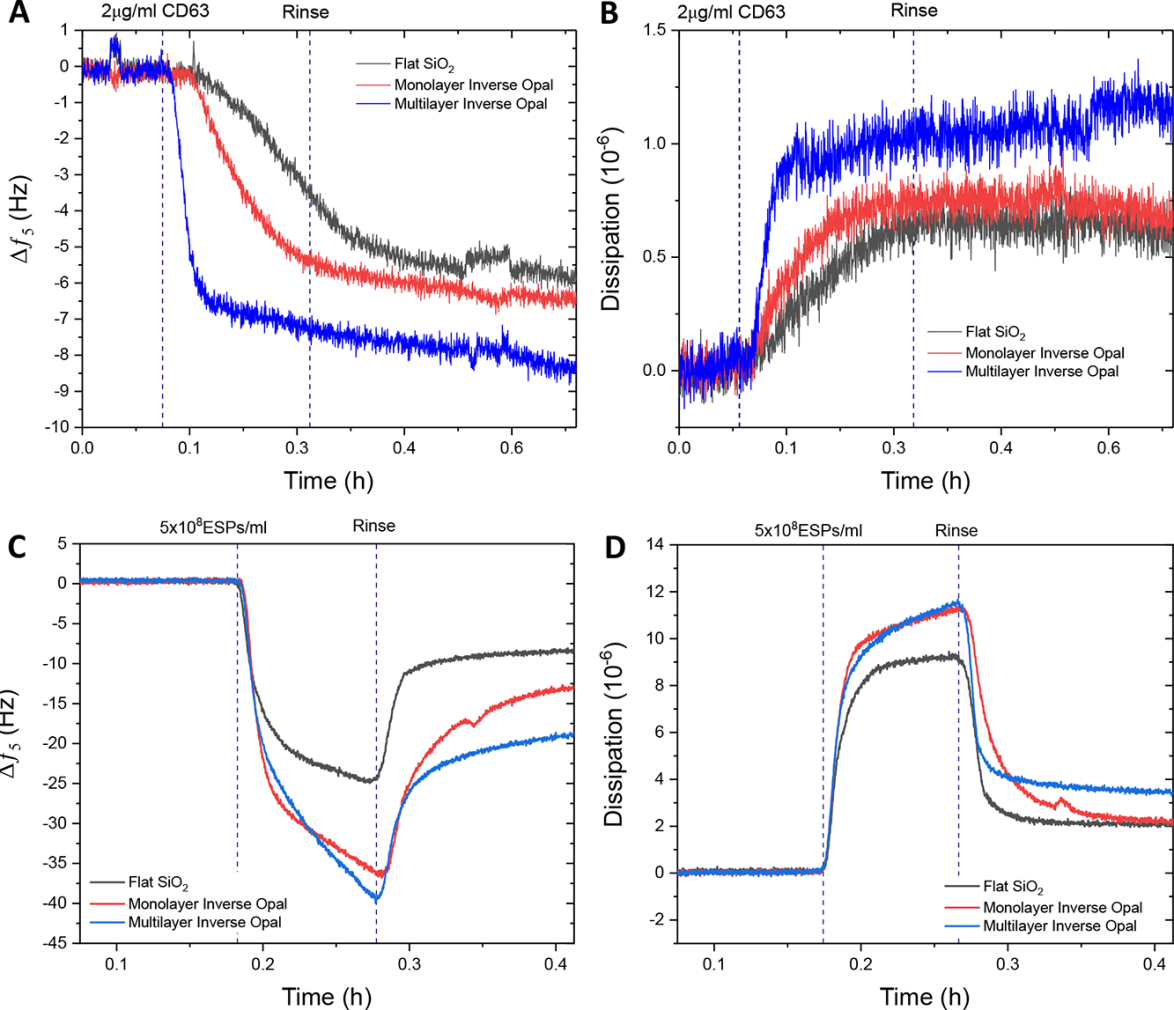
Comparison of frequency (A) and dissipation (B) profiles for 2 μg/mL CD63 detection on flat silica, inverse opal monolayer, and multilayer surfaces. QCM-D analysis of EV detection using IO immunofunctionalized sensors. QCM-D frequency (C) and dissipation (D) profiles comparing responses to CD63-positive EVs in 25% v/v human serum of flat silica, silica inverse opal monolayer, and multilayer surfaces.

After functionalization, the performance of all three surfaces was assessed against a spiked concentration of CD63 protein. Figure 5A, B shows that an incremental increase in response occurs with the increasing internal surface area of the structure. The monolayer exhibited a marginal improvement in CD63 detection compared to the flat silica, while the multilayer IO increased the response further by approximately 30% in terms of frequency change. Dissipation changes upon the addition of CD63 were small in nature, which was expected due to the largely nonviscoelastic properties of the spiked protein. This suggests that the protein was able to bind rigidly to the IO structure, irrespective of the layer thickness. Spiked CD63 is a small molecule (2.4 nm) compared to the pore size, and thus would not induce any pore blockage but rather likely infiltrate the entire IO structure through the interpore necks.

The detection of the considerably larger, fluid-filled, ESP structures on these surfaces was explored via the addition of 5 × 10^8^ ESPs/mL in 25% v/v serum (Figure 5C, D). The net decrease in frequency following a post-adsorption rinse signified the adsorption of particles to all three sensor surfaces, the most significant of which was seen with the multilayer IO structure, offering almost a 100 and 40% increase in response compared to flat and monolayer IO surfaces, respectively (Figure 5C). The corresponding improvement in dissipation was also detected (Figure 5D). Further analysis of the adsorption and desorption of EVs on flat SiO_2_ vs multilayer IO is shown in the Supporting Information. A linear fit of the initial phase of mass uptake and release based on the response of the frequency channel (Figure S4) provides an enhanced rate of adsorption of −0.46 Hz s^−1^ for the multilayer IO structure in comparison to −0.36 Hz s^−1^ for flat SiO_2_. The rate of desorption was comparable with 0.21 Hz s^−1^ for the multilayer IO and 0.21 Hz s^−1^ for the flat SiO_2_, respectively. The adsorption and desorption kinetics of the process were further investigated across a longer timescale to obtain characteristic time constants (Figure S5). Herein, a mass viscoelastic model was used for quantitative comparison.^56^ As expected, the mass uptake of the flat SiO_2_ sensor could be fitted with a single exponential function, resulting in a τ_ad_ of 41 s. In contrast, the mass uptake of the multilayer IO sensor did not follow one growth rate constant throughout. The process can be better approximated with a bi-exponential fit, resulting in τ_ad,1_ of 25 s and τ_ad,2_ of 417 s. This may be explained using a two-stage adsorption process. Initially, the multilayer IO provides an enhanced surface area for EV anchoring. However, with every binding event, the number of percolation paths for further uptake is reduced in the 3D architecture, requiring more complex diffusion processes for additional mass uptake. (Note that with a bi-exponential fit, the corresponding values for flat SiO_2_ were τ_ad,1_ = 30 s and τ_ad,2_ = 87 s.) Importantly, the desorption process was comparable for both flat SiO_2_ and multilayer IO, with τ_des, flat_ of 45 s and τ_des, IO_ of 48 s, respectively. These findings provide further evidence that the 3D architecture was well accessible for EVs.

**Figure 6:**
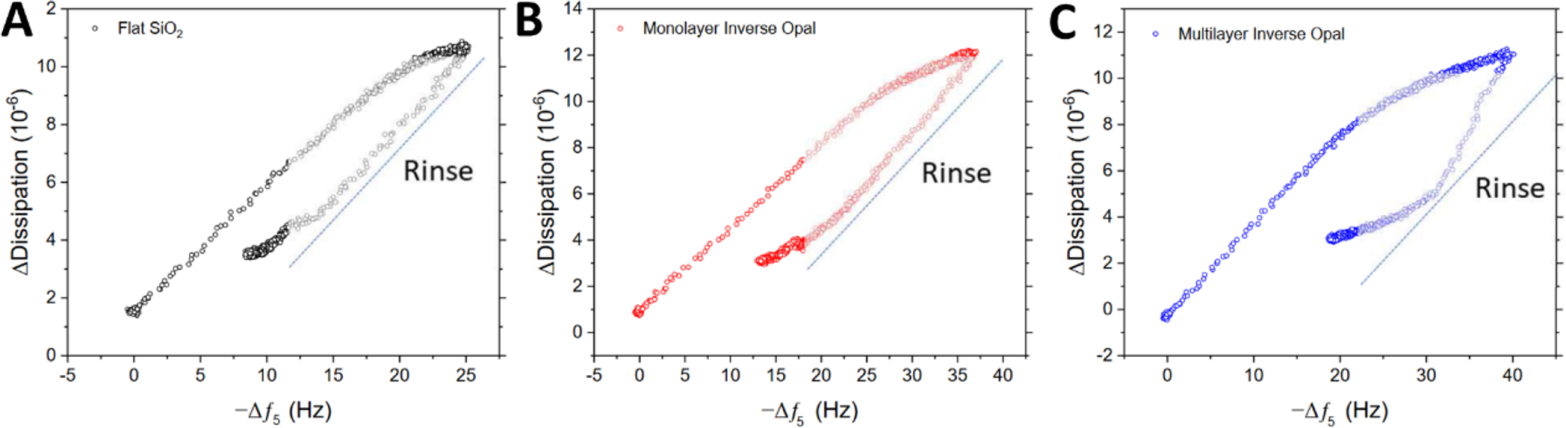
Comparing dissipation response as a function of frequency change across inverse opal functionalized surfaces. Response analysis using (A) flat silica, (B) silica inverse opal monolayer, and (C) silica inverse opal multilayer substrates toward 1 × 10^9^ ESPs/mL in 25% v/v serum.

To gain more insights into the ESP binding process across the different architectures, dissipation change was assessed as a function of frequency for ESP adsorption and rinse steps (Figure 6). All three sensor surfaces demonstrated an initial linear relationship between dissipation and frequency as ESPs were captured, with a subsequent decrease in the relative dissipation response as the surface nears saturation. The follow-up rinsing of the sensor surface resulted in significant frequency and dissipation reduction for both flat and monolayer IO surfaces, indicating comprehensive removal of many loosely bound, or nonspecifically adsorbed, artifacts from the sensing surface. These are assumed to be the serum content of the running buffer. In contrast, the multilayer IO sensor exhibited a stunted removal of such artifacts and a prolonged period of frequency reduction alongside a minimal decrease in dissipation. This may be a result of the continual removal of surface-bound contaminants, albeit to a lesser extent. Moreover, the thickness and interconnectivity of the multilayer IO could result in the entrapment of artifacts and small non-ESP particles within the porous network, which contributed to the elevated dissipation signal.

To substantiate this theory, control investigations were conducted by flowing 1 × 10^9^ ESPs/mL in HEPES buffered saline (HBS) buffer and 25% v/v serum across the three surface types following functionalization with a nonspecific IgG control antibody to determine the background nonspecific binding contribution to the overall response. Figure S6 indicates that the introduction of the sample to the sensor surface caused a small yet significant response in frequency and dissipation for multilayer IO surfaces (particularly in a more complex media), while monolayer IOs demonstrated negligible change. It is possible that the multilayer architecture entrapped a greater volume of the sample, increasing the oscillatory mass but decreasing the layer rigidity and pathways for energy dissipation. Responses seen with multilayer IOs should therefore be approached with caution along with the knowledge of buffer composition.

**Figure 7:**
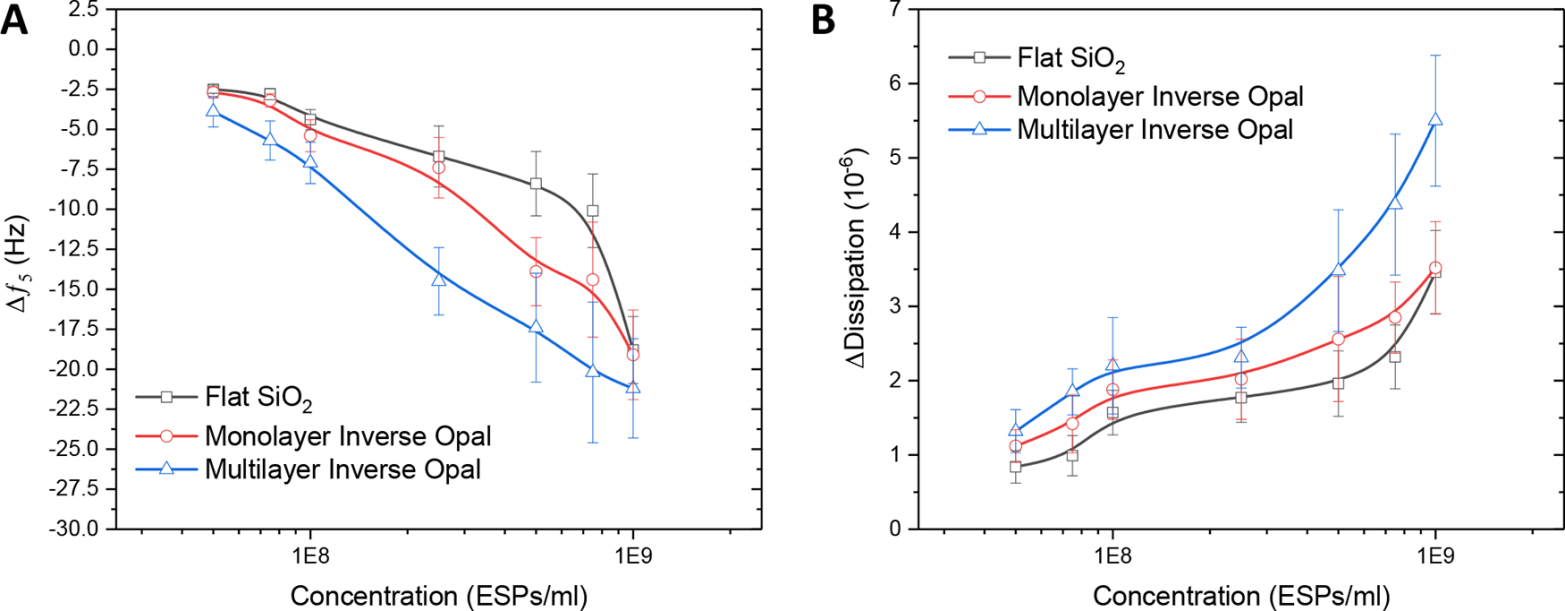
QCM-D performance comparison between flat silica, silica inverse opal monolayer, and multilayer surfaces against titrated concentrations of ESPs spiked in 25% v/v serum. QCM-D (A) frequency and (B) corresponding dissipation profiles. Standard deviation is determined from three independent experiments.

Subsequently, the sensing performance of the IO architectures was explored across a range of ESP concentrations to determine the impact on detection sensitivity. Data for both frequency and dissipation response are shown in Figure 7. Interestingly, multilayer IOs seemed to exhibit stronger responses across all tested ESP concentrations for both methods of measurement, as well as increasing the dynamic range of detection compared to flat silica. It is likely that the increased internal surface area of the IO supports additional antibody functionalization and subsequent EV capture. It is also apparent that frequency responses for all three surfaces converged at the highest concentration of ESPs (Figure 7A). Conversely, the dissipation response for multilayer IOs continued to increase and at a faster rate than for the flat silica or monolayer IO (Figure 7B).

Table 1 compares the LOD of the silica-based sensors. IO structures were shown to improve detection performance toward ESPs, with high surface area multilayer IOs demonstrating a frequency and dissipation LOD as low as 6.24 × 10^7^ and 6.91 × 10^7^ ESPs/mL, respectively. These results should be evaluated alongside the findings of potential artifacts and solvent entrapment for the multilayer 3D IOs compared to the monolayer 2D IOs. Thus, the observed improvement in performance is somewhat limited. The sensing characteristics compare favorably to some reports adopting spectroscopic principles, which,^57,58^ however, would benefit from further advancement when comparing plasmon resonance-based approaches, exhibiting LODs as low as 1 *×* 10^3^ ESPs/mL.^59^

**Table 1:** QCM-D LOD Values Calculated for the Different Silica Surfaces Used during This Work

	LOD vs sensor surface type		
mode	flat silica	IO monolayer	IO multilayer
frequency	9.60 × 10^7^	9.24 × 10^7^	6.24 × 10^7^
dissipation	9.01 × 10^7^	8.42 × 10^7^	6.91 × 10^7^

Nonetheless, the combination of highly tunable, scalable, and low-cost fabrication of the IO structures underlines their potential to comprise a reliable method for improving QCM-D analytical performance.

## Conclusions

This work investigated the potential of inorganic IO networks for improved QCM-D biosensing performance. Vertical withdrawal and evaporative deposition techniques were employed as two co-assembly methods to produce silica IOs with monolayer (2D) and multilayer (3D) thickness, respectively. SEM characterization confirmed some degree of shrinkage from the original colloidal sphere size, resulting in the formation of oblate ellipsoidal pores. This allowed the internal surface area to be estimated as being 2-fold and 43-fold larger for monolayer and multilayer IOs, respectively, compared to a flat silica surface. The generation of these structures on silica QCM sensors supported successful immunosensing of spiked CD63 protein, followed by CD63-positive EVs. IO structures were shown to improve detection performance toward ESPs, with high surface area multilayer IOs demonstrating a frequency and dissipation LOD as low as 6.24 × 10^7^ and 6.91 × 10^7^ ESPs/mL, respectively. However, possible findings of the solvent and artifact entrapment within the 3D IO structures suggest that careful investigation of such systems is needed to validate their effectiveness. 2D IO surfaces, on the other hand, offer the scope for compartmentalized ligand and analyte anchoring. With the reusability of these sensors previously shown,^14^ further fabrication and sensing strategies will create opportunities for advanced dual-mode analysis of clinically relevant biomarkers by combining QCM-D with optical methods that exploit the photonic band gap properties of the IO architectures.

## Experimental Section

### EV Isolation and Characterization

SEC isolation of 10 × 1 mL ESPs fractions from human mesenchymal stem-cell cell-culture media (HUMSCCM) was conducted, as detailed in previous studies.^17^ Briefly, 30 mL of the clarified media obtained after filtering the HUMSCCM source with a 0.45 μm filter (Merck Millipore, U.S.) was subsequently concentrated via centrifugation at 4000 g for 30 min at 4 °C, using Amicon Ultra-15 centrifugal filters with a 10 kDa pore size cut-off (Merck Millipore, USA). In a next step, 0.5 mL of the obtained concentrated solution was loaded onto a qEV 35 nm SEC column (Izon Science, UK) and eluted at a flow rate of 1 mL/min, using an HBS (0.01 M HEPES, pH 7.4, 0.15 M NaCl) (GE Healthcare Life Sciences, Sweden). Collected SEC fractions were characterized using NTA analysis (Nanosight LM10 instrument, Malvern Instruments, UK) and western blot analysis (Biotechne Ltd., USA), as described in a previous study.^14^

### Colloidal Suspension Preparation

A TEOS mixture consisting of 1:1:1.5 ratio (by weight) of TEOS, 0.10 M HCl, and EtOH (100%) was prepared. This mixture (0.15 mL) was added to 19.5 mL of deionized water and 0.5 mL of a 5% w/v colloidal PMMA particle (250 and 600 nm diameter) suspension in water (predispersed by sonication). The solution was stirred for 1 h at room temperature prior to use.

### Monolayer Formation via Vertical Withdrawal Co-Assembly

Bare Si wafers (1 × 2 cm) and silica-coated QCM sensors were exposed to 60 s of oxygen plasma (20 sccm) using a Diener Electronic PICO instrument to remove organic contaminants and for oxide activation to introduce desirable hydrophilic properties for co-assembly. Si wafers and silica-coated QCM sensors were suspended in a container of colloid/TEOS suspension using a custom-made motorized sample holder. The submerged sample was withdrawn at a programmed rate of 0.01 mm/min over 24 h, inducing thin film deposition at the air-solvent interface. Post-deposition, wafers/sensors were annealed at 180 °C for 2 h to aid mechanical stability prior to removal of PMMA. The PMMA opal template was removed by oxygen reactive ion etching using oxygen plasma exposure for 300 s (20 sccm).

### Multilayer Formation via Vertical Evaporative Deposition Co-Assembly

As before, bare Si wafers (1 × 2 cm) and silica-coated QCM sensors were exposed to 60 s of oxygen plasma (20 sccm) using a Diener Electronic PICO instrument. Si wafer and silica-coated QCM sensors were then suspended in a container of colloid/TEOS suspension using a custom-made sample holder. The colloidal suspension was evaporated over a 2-day period in a 65 °C oven, inducing film deposition at the air-solvent interface. Post-deposition, wafers/sensors were annealed at 180 °C for 2 h to aid mechanical stability prior to removal of PMMA. The opal substrates were then calcined in air at 500 °C for 2 h with a 4 h ramp time for the removal of PMMA and sintering of the silica inverse opal structures.

### Scanning Electron Microscopy

Si wafers with inverse opal films were analyzed with SEM using a JEOL 6701 instrument (Japan). All micrographs were collected at an accelerating voltage of 10 kV. Samples were mounted for both cross-sectional and longitudinal imaging on black carbon tape followed by gold sputter coating for 10 s at 0.08 mBarr prior to analysis. In-plane and out-of-plane pore size distributions were determined using the Pebbles software.^53^

### Grazing Incidence Small-Angle X-ray Scattering

GISAXS experiments were performed at the Centre for Nature Inspired Engineering (University College London), using a SAXSLab Ganesha 300XL (8 keV) with an incident angle of 0.18°. 2D scattering patterns were collected with a PILATUS 300 K detector with a sample-to-detector distance of 1400 mm. GISAXS data analysis was performed using FitGISAXS software.^60^ Si wafers with inverse opals formed from 100 nm PMMA spheres using the evaporative deposition approach were used for analysis to understand the structural order of the pores.

### General QCM Apparatus Setup

A Q-Sense E4 QCM-D instrument (Biolin Scientific, Sweden), coupled with the QTools software (version 3.0.17.560, Biolin Scientific, Sweden), was used to perform and analyze the QCM-D measurements. All changes in resonance frequency (*Δf*) presented here correspond to the one recorded from the fifth overtone because of its optimal signal stability-sensitivity ratio. Variations of less than 10% in Δ*f* were observed between all the registered overtones (3th, 5th, 7th, and 11th, respectively). In line with previous studies, samples were degassed prior to exchange in the QCM-d flow module, and AT-cut 5-MHz gold-coated quartz crystal sensors with a 0.79 cm^2^ active area (Biolin, Sweden) were used. The impact of buffer properties on the sensing process was minimized by using the same HBS stock solution during the preparation of all analyte solutions, ensuring maximum reproducibility and allowing direct comparison between the Δ*f* detected. In a subsequent step, identical volumes (0.25 mL per sensor) of the prepared analyte solutions were flowed at 10 μL/min over the sensor surfaces. Baseline measurements were routinely performed simultaneously to the sample measurements using a bare sensor. Frequency and dissipation values reported here are postbuffer rinse to account for the removal of weakly bound analytes. All experiments were conducted at least in triplicate with representative QCM-D profiles depicted in Figures 4 and 5.

### Silica-Coated QCM Sensor Functionalization

A 5 mM solution of silane-PEG (2 kDa)-biotin and spacer molecule silane-PEG (600 Da)-COOH at a 1:9 molar ratio was flowed across the sensor surface at 7.5 μL/min overnight to form a mixed SAM. Ratios were determined following optimization studies using molecules of a similar size in previous reports.^14^ A 100 μg/mL solution of SAv was flowed across the sensor surface at 10 μL/min for 20 min, followed by a rinse step of HBS at 80 μL/min for 15 min. Mouse monoclonal biotinylated-anti CD63 (20 μg/mL) was immobilized on the surface at 10 μL/min for 20 min, followed by another rinse step and response stabilization for 30 min prior to sample addition.

### QCM-D Immunodetection of EVs Using Silica Sensors

Silica-based immunosensor functionality toward spiked CD63 and exosomal CD63 was assessed. Spiked samples of CD63 tetraspannin protein with a concentration of 35 nM were used as a positive control. Sensitivity toward CD63-positive EVs in HBS buffer was tested using dilutions of SEC fraction 4. ESP samples were titrated in 25% v/v human serum (Sigma Aldrich, USA) to determine the sensitivity of the platform in complex media. The sensors were assessed with the following concentrations: 5 × 10^7^, 7.5 × 10^7^, 1 × 10^8^, 2.5 × 10^8^, 5 × 10^8^, 7.5 × 10^8^, and 1 × 10^9^ ESPs/mL. The specificity of the sensor surfaces and the background signal were determined by replacing the anti-CD63 antibody with a biotin-IgG isotype control antibody. Responses between sensor surfaces were compared using a concentration of 1 × 10^9^ ESPs/mL in HBS buffer and 25% v/v serum. LOD and LOQ were defined as the minimum concentration displaying a signal-to-noise ratio of 3 and 10, respectively.^61^ SNR was determined as a ratio of the response elicited on the target and control sensor surfaces.

## Supporting Information Available

Graphical images of the sample holders used for the fabrication of the functionalized QCM-D sensors; GISAXS characterization of the multilayer inverse opal films; pore size distribution from SEM images; fits of the mass uptake and release during QCM-D sensing; and QCM-D response of nonspecific sensors (PDF)
