# Supplementary material for: Silica Inverse Opal Nanostructured Sensors for Enhanced Immunodetection of Extracellular Vesicles by Quartz Crystal Microbalance with Dissipation Monitoring
Source: ACS Appl Nano Mater. 2022 Aug 19;5(9):12951–61. doi: 10.1021/acsanm.2c02775 (PMC9513796; doi:10.1021/acsanm.2c02775)
Supplement: Supplementary file 1 — an2c02775_si_001.pdf [file an2c02775_si_001.pdf]

-Supporting Information-

Silica Inverse Opal Nanostructured Sensors for Enhanced Immunodetection of Extracellular Vesicles by Quartz Crystal Microbalance with Dissipation Monitoring

Jugal Suthar,[1,2] Alberto Alvarez-Fernandez,[1,] Alaric Taylor,[1] Maximiliano J. Fornerod,[1] Gareth R. Williams,[2] and Stefan Guldin.[1,*]*

[1]Department of Chemical Engineering, University College London, Torrington Place, London, WC1E 7JE, United Kingdom.

[2]UCL School of Pharmacy, University College London, 29-39 Brunswick Square, Bloomsbury, London, WC1N 1AX, United Kingdom.

Email: alberto.fernandez@ucl.ac.uk; s.guldin@ucl.ac.uk

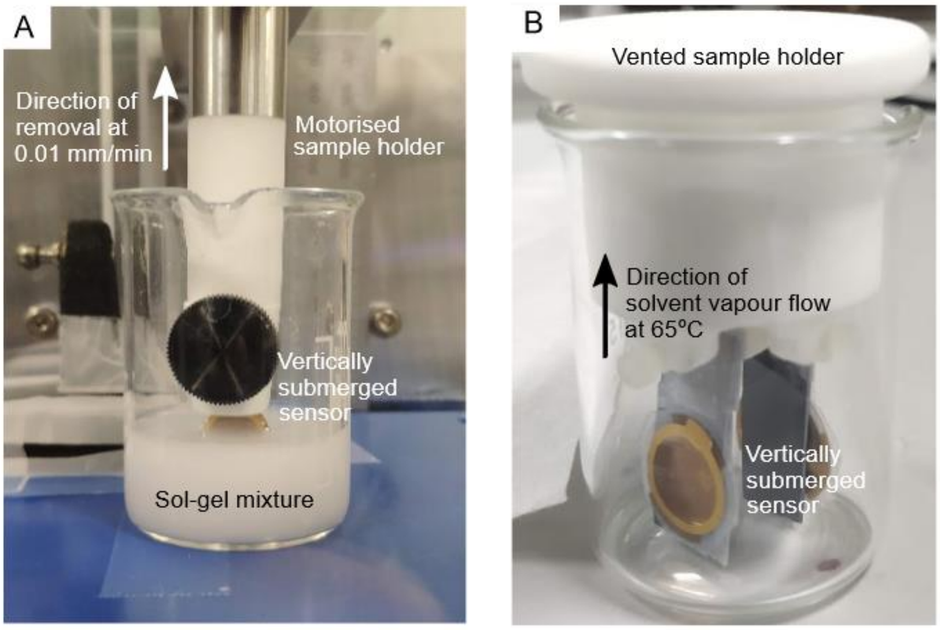

Figure S1: Sample holders utilised in colloidal co-assembly fabrication process. (A) Vertical withdrawal and (B) evaporative deposition apparatus set-up.

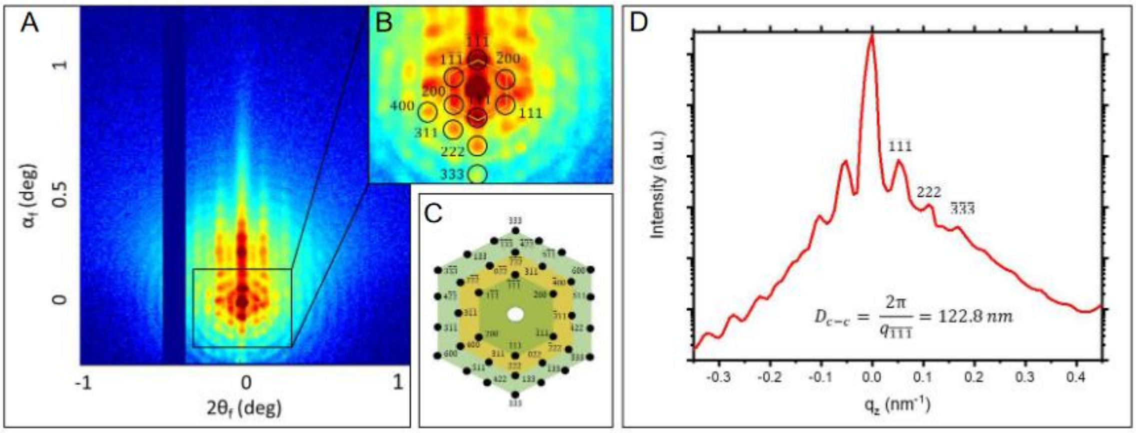

Figure S2: GISAXS characterisation of multilayer inverse opal. (A) 2D GISAXS scattering diffraction pattern and (B) detailed view of multilayer inverse opal structure created from 100 nm PMMA spheres. (C) Simulated diffractograms for the 2D projection of a FCC lattice of scatterers along the {111} plane. (D) A vertical (in-plane) line-cut profile taken as a function of qz.

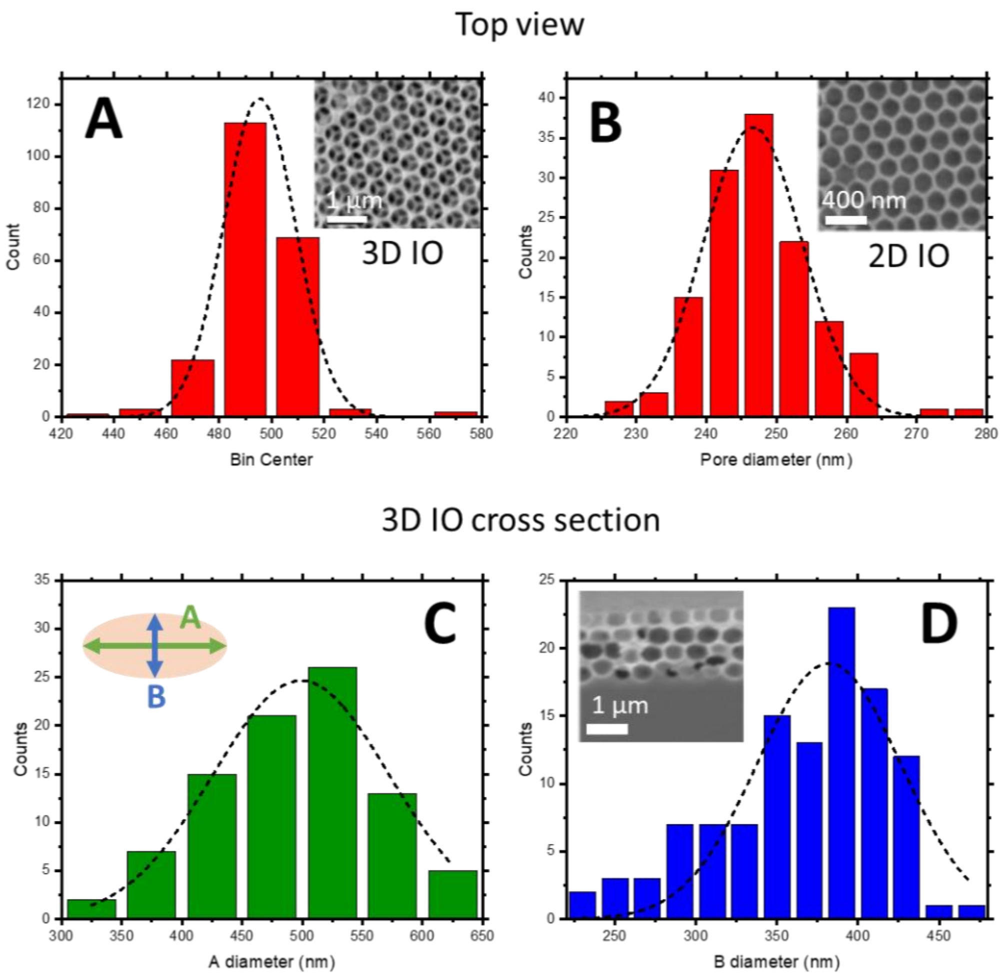

Figure S3: Pore size distribution obtained from image analysis of top-view SEM images of IO multilayer (A) and monolayer (B). Pore size determination of the ellipsoidal pores of the IO multilayer architecture from cross-sectional SEM image: width (C) and height (D)

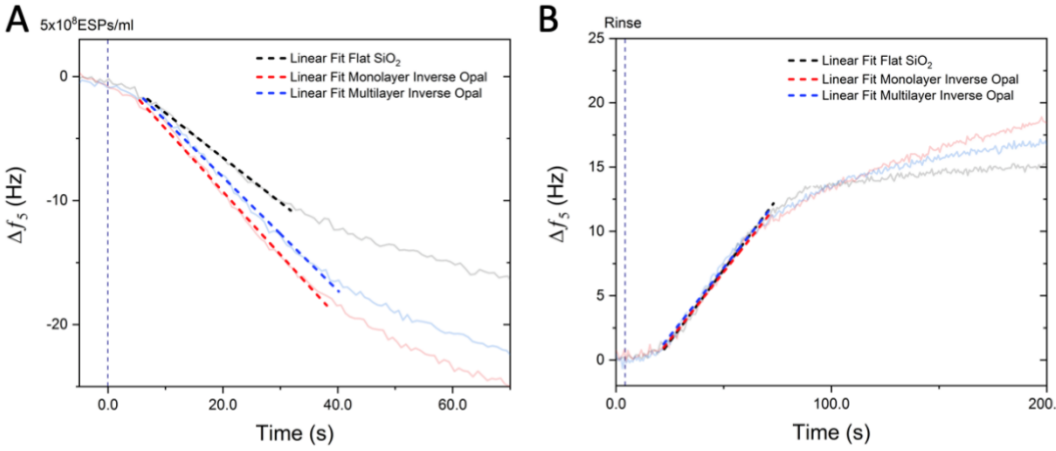

Figure S4: Linear fit of the initial phase of mass uptake (A) and release (B) based on the response of the frequency channel (see Figure 5C).

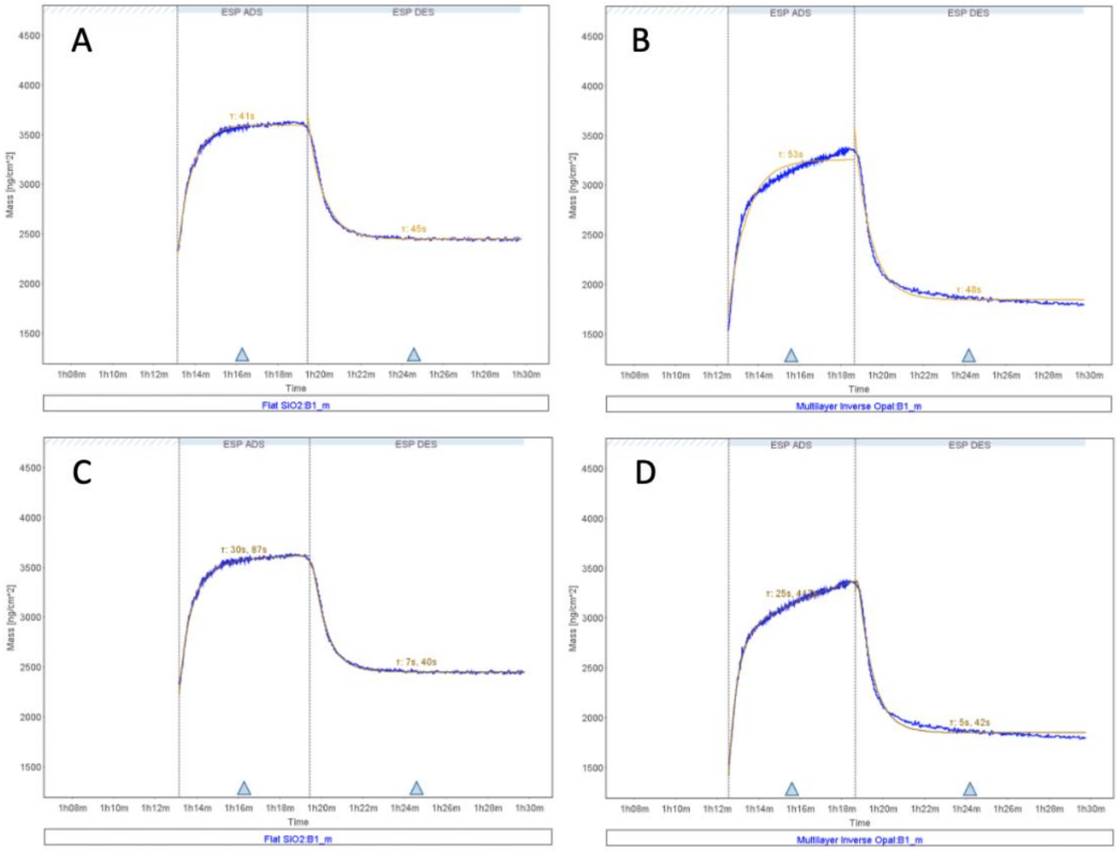

Figure S5: Exponential fit of mass uptake and release for flat SiO_2 (A.B) and multilayer IO (C,D). Note that the fits in A and C are based on a single exponential fit, while B and D are based on a biexponential function to represent two distinct growth and decay processes.[1]

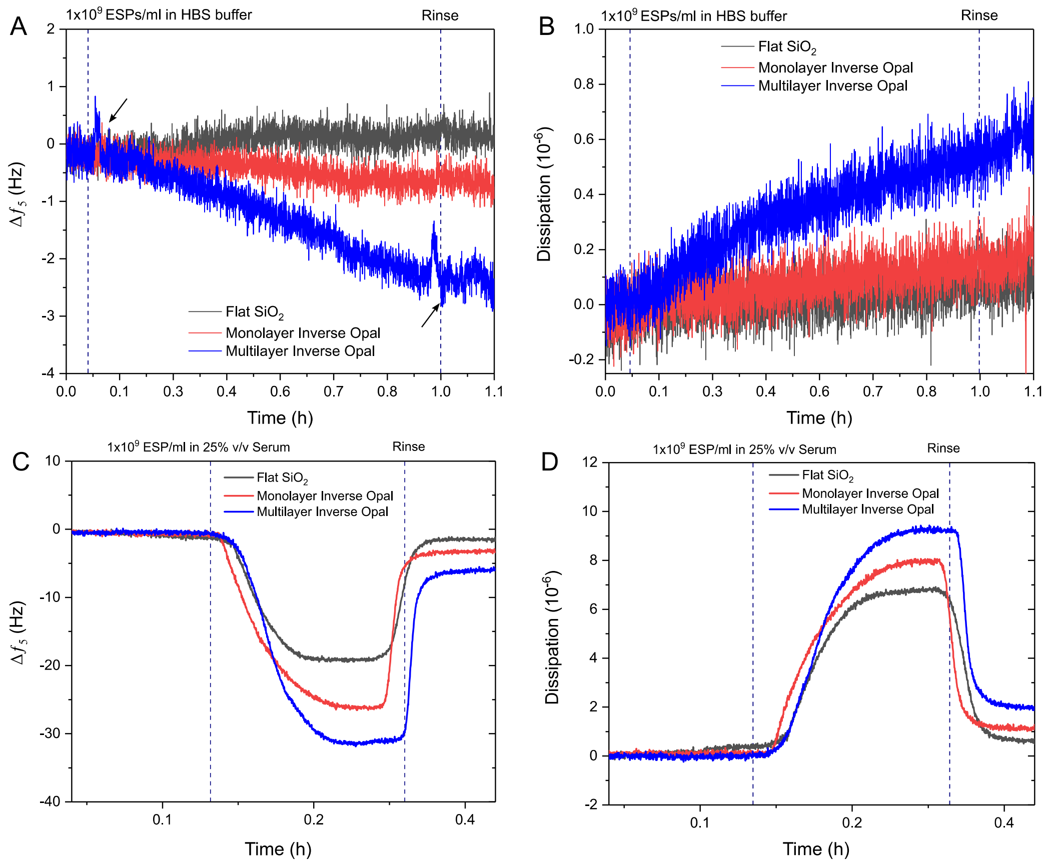

Figure S6: QCM-D response comparison using control 3DIO sensors against CD63-positive EVs. Example (A) frequency and corresponding (B) dissipation profiles acquired upon addition of 1x10^9 ESPs/mL in HBS buffer to control non-specific sensor. Black arrows indicate signal disturbances attributed to presence of air bubbles within the sample chamber. Example (C) frequency and corresponding (D) dissipation profiles acquired upon addition of 1x10^9 ESPs/mL in 25\% v/v serum to control non-specific sensor. Example QCM-D profiles taken from 3 replicates.

References

(1) Voinova, M. v; Rodahl, M.; Jonson, M.; Kasemo, B. Viscoelastic Acoustic Response of Layered Polymer Films at Fluid-Solid Interfaces: Continuum Mechanics Approach. *Physica Scripta* **1999**, *59* (5), 391–396. https://doi.org/10.1238/PHYSICA.REGULAR.059A00391/XML.
